# Human papillomavirus infection and other risk factors for cervical intraepithelial neoplasia in Japan

**DOI:** 10.1038/sj.bjc.6690401

**Published:** 1999-05-01

**Authors:** H Yoshikawa, C Nagata, K Noda, S Nozawa, A Yajima, S Sekiya, H Sugimori, Y Hirai, K Kanazawa, M Sugase, H Shimizu, T Kawana

**Affiliations:** Department of Obstetrics and Gynecology, University of Tokyo, Hongo 7-3-1 Tokyo, 113-8655, Japan; Department of Public Health, Gifu University, Gifu,Japan; Department of Obstetrics and Gynecology, Kinki University, Osaka, Japan; Keio University, Tokyo, Japan; Tohoku University, Sendai, Japan; University of Chiba, Chiba, Japan; Saga Medical School, Saga, Japan; Cancer Institute Hospital, Tokyo, Japan; University of Ryukyu, Okinawa, Japan; Nagano Red Cross Hospital, Nagano, Japan; Tokyo University Branch Hospital, Tokyo, Japan

**Keywords:** cervical intra-epithelial neoplasia, human papillomavirus, case-control study, risk factors

## Abstract

Various risk factors were investigated in 167 cervical intra-epithelial neoplasia (CIN) case and control pairs in Japan. CIN cases showed evidence of nine known risk factors including smoking and sexual behaviour. However, after adjustment for papillomavirus infection, the highest determinant, the only remaining risk factors were: being married, early age at first pregnancy and multiparity. © 1999 Cancer Research Campaign


British Journal of Cancer (1999) 80(3/4), 621–624
© 1999 Cancer Research Campaign
Article no. bjoc.1998.0401

### Human papillomavirus infection and other risk factors for cervical intraepithelial neoplasia in Japan

H Yoshikawa[1], C Nagata[2], K Noda[3], S Nozawa[4], A Yajima[5], S Sekiya[6], H Sugimori[7], Y Hirai[8], K Kanazawa[9], M Sugase[10], H Shimizu[2] and T Kawana[11]

[1]Department of Obstetrics and Gynecology, University of Tokyo, Hongo 7-3-1 Tokyo, 113-8655, Japan; [2]Department of Public Health, Gifu University, Gifu, Japan; [3]Department of Obstetrics and Gynecology, Kinki University, Osaka, Japan, [4]Keio University, Tokyo, Japan, [5]Tohoku University, Sendai, Japan, [6]University of Chiba, Chiba, Japan, [7]Saga Medical School, Saga, Japan, [8]Cancer Institute Hospital, Tokyo, Japan, [9]University of Ryukyu, Okinawa, Japan, [10]Nagano Red Cross Hospital, Nagano, Japan, [11]Tokyo University Branch Hospital, Tokyo, Japan

**Summary** Various risk factors were investigated in 167 cervical intra-epithelial neoplasia (CIN) case and control pairs in Japan. CIN cases showed evidence of nine known risk factors including smoking and sexual behaviour. However, after adjustment for papillomavirus infection, the highest determinant, the only remaining risk factors were: being married, early age at first pregnancy and multiparity.

**Keywords:** cervical intra-epithelial neoplasia; human papillomavirus; case-control study; risk factors

It is widely accepted that human papillomavirus (HPV) is the primary causative agent of cervical neoplasia (cervical intraepi thelial neoplasia [CIN] and invasive cancer) (zur Hausen, 1991). However, HPV infection of the uterine cervix does not always induce cellular abnormalities (de Villiers et al, 1987), suggesting that other factors play a role in the development of cervical neoplasia. Many epidemiologic factors have been suggested as relevant to the development of cervical neoplasia (Rotkin, 1973; Bornstein et al, 1995). We conducted a case-control study to clarify how epidemiologic factors are involved in the development of CIN in relation to HPV infection.

### MATERIALS AND METHODS

### Study population

The study population consisted of women aged 55 or younger who underwent Papanicolaou test screening at nine hospitals between June 1995 and July 1996. For women with abnormal cervical cytology, colposcopically directed biopsies were performed. The cases enrolled in the study were women in whom CIN was first detected during the study period. A total of 167 women who had histological evidence of CIN were included; low-grade CIN (CIN I [$n = 94$]) and high-grade CIN (CIN II [$n = 40$] and CIN III [$n = 33$]). We excluded carcinoma in situ from CIN III. The histological review was performed by one experienced pathologist (KN). Controls matched one-to-one with cases on age (within 5 years) and hospital were selected from subjects who were found to have normal cervical cytology. All CIN cases and controls gave written informed consent.

*Received 30 September 1998*
*Revised 13 November 1998*
*Accepted 19 November 1998*

*Correspondence to:* H Yoshikawa

### The questionnaire

Information concerning health issues related to the possible aetiology of CIN was obtained by a self-administered questionnaire. The questionnaire, which included demographic factors, smoking habits, contraceptive and reproductive history, and sexual behaviour, was distributed to each case or control on their second hospital visit. Several participants gave no answers to certain questions.

### Detection and typing of HPV

Cellular DNA was extracted from cervical exfoliated cells by standard procedure. We used the consensus L1 primers, L1C1 (1 μM), L1C2 (0.5 μM) and L1C2M (0.5 μM), for the polymerase chain reaction (PCR) (Yoshikawa et al, 1991). HPV types were identified on the basis of the restriction fragment length polymorphism. The assay can type at least 26 genital HPVs. HPV data were not obtained from nine (5%) of CIN cases and 37 (22%) of controls. HPV types were categorized into three groups of carcinogenic potency according to then risk classification proposed by Lorincz et al, (1992); i.e. high-, intermediate- and low-risk HPVs (Table 1).

### Blood sampling and serum antibody detection

A blood sample for serological assays of herpes simplex virus (HSV), cytomegalovirus (CMV) and *Chlamydia trachomatis* (CT) was collected from each subject. Blood sampling was not available in 9 (5%) CIN cases and 5 (3%) controls. Antibodies were assayed by using commercially available enzyme-linked immunosorbent assay (ELISA) kits; HSV and CMV (Denkaseiken, Niigata, Japan), and CT (Labsystems Oy, Helsinki, Finland).

### Data analysis

Odds ratios (ORs) and the corresponding 95% confidence intervals (Cls) were calculated for each variable using conditional

**Table 1** Detection of HPV DNA in controls and CIN cases

| | High risk | | Intermediate-risk | | | | | | | | | Low risk | | | | | | | | |
|---|---|---|---|---|---|---|---|---|---|---|---|---|---|---|---|---|---|---|---|---|
| **Lesions** | **16** | **18** | **31** | **33** | **51** | **52** | **56** | **58** | **59** | **68** | **70** | **6** | **42** | **53** | **54** | **61** | **66** | **X[a]** | **U[b]** | **HPV + (%)** |
| Controls | 1 | 1 | 2 | 0 | 1 | 5 | 2 | 1 | 1 | 0 | 1 | 0 | 0 | 1 | 0 | 1 | 0 | 2 | 0 | 19/130 (14.6) |
| Low-grade CIN | 6 | 0 | 0 | 1 | 7 | 10 | 4 | 10 | 0 | 3 | 3 | 0 | 1 | 0 | 1 | 0 | 1 | 7 | 6 | 60/86 (69.8) |
| High-grade CIN | 12 | 2 | 3 | 4[c] | 4[d] | 12 | 1 | 6[cd] | 1 | 1 | 0 | 1 | 0 | 1 | 1 | 0 | 0 | 6 | 8 | 61/72 (84.7) |

[a]Unknown HPV, [b]type-undertermined HPV, [c]dual infection in one case, [d]dual infection in another case.

**Table 2** Relative risks (ORs) of CIN according to HPV test results

| | Controls | CIN | | High-grade CIN II and III | |
|---|---|---|---|---|---|
| **HPV test results** | **No. (%)** | **No. (%)** | **OR (95% CI)** | **No. (%)** | **OR (95% CI)** |
| Negative | 111 (85.4) | 37 (23.4) | 1.0 | 11 (15.2) | 1.0 |
| Positive | 19 (14.6) | 121 (76.6) | 30.0 (9.5–94.8) | 61 (84.7) | 46.0 (6.3–333.6) |
| High-risk HPVs | 2 (1.5) | 20 (12.7) | 109.6 (9.1–1320) | 14 (19.4) | 68.5 (4.1–1139) |
| Intermediate-risk HPVs | 13 (10.0) | 68 (43.0) | 19.8 (6.1–64.4) | 30 (41.6) | 36.5 (4.2–316.2) |
| Low-risk HPVs | 4 (3.1) | 19 (12.0) | 41.5 (6.1–283.6) | 9 (12.5) | 22.4 (1.8–270.0) |

Type-undetermined HPV was excluded from the risk classification of HPV.

logistic regression technique. Continuous variables were categorized to allow ORs to be computed. Alongside crude ORs, we calculated ORs after adjustment for HPV infection (HPV-adjusted ORs). HPV infection and HPV-independent variables were assessed by multivariate analysis. Tests for linear trend were mainly performed on continuous variables. All the above-mentioned statistical analyses were done using the SAS computer software package (SAS Institute, Cary, NC, USA). Association between two variables was analysed using a Spearman rank correlation coefficient.

### RESULTS

The mean ages of CIN cases and controls were 40.3 years and 40.7 years respectively. The age distribution was almost the same in the two groups.

### HPV infection

HPV DNA was detected much more frequently in CIN cases than controls (Tables 1 and 2, OR = 30.0). The incidence and types of HPV detected in CIN cases and controls are shown in Table 1. In the case of CIN as a whole, and high-grade CIN, no significant differences in ORs were found across the three HPV subgroups with different carcinogenic potentials (Table 2).

### Risk factors before and after adjustment for HPV infection

Of 16 study variables, the following nine showed statistically significant differences between cases and controls before adjustment for HPV infection: smoking, being married, early age at marriage, multiple pregnancies, multiparity, early age at first pregnancy, multiple sexual partners, positive CT IgA and positive CT IgG (Tables 3 and 4). Most of these risk factors were no longer significant after adjustment and, in particular, the elevated risks associated with smoking and multiple sexual partners disappeared completely (HPV-adjusted OR $< 1.0$). In contrast, the associations between being married, multiparity and early age at first pregnancy with CIN risk remained significant after adjustment.

The multivariate analysis for HPV infection and the three HPV-independent variables confirmed that these factors were independently and significantly associated with the risk of CIN (HPV infection, OR = 113; currently being married, OR = 8.75, three or more births, OR = 4.00; 23 years or younger at first pregnancy OR = 7.79).

### DISCUSSION

The present study confirmed that the highest risk determinant for CIN was HPV infection. As expected, CIN patients had various habitual, reproductive and sexual risk factors. However, these factors, except for multiparity, early age at first pregnancy and being married, were no longer significant after adjustment for HPV infection.

The independent role of multiparity for development of CIN in our study is of particular interest. Several recent studies controlling for HPV infection have also observed an association between multiparity and risk of CIN (Schiffman et al, 1993; Becker et al, 1994). It was reported that progesterone enhances expression of E6 and E7 oncoproteins through progesterone-responsive elements of HPV (Chen et al, 1996). In addition, there is a recent report suggesting that progesterone may impair T-cell recognition of HPV-infected cells (Bartholomew et al, 1997). It seems likely that higher levels of progesterone derived from the placenta may be associated with CIN development.

Early age at first pregnancy has not been consistently shown to be a risk factors in the previous studies. As expected, this factor was closely correlated with early age at first birth (Spearman rank correlation coefficient = 0.87, $P = 0.0001$). In a British study,

 *© Cancer Research Campaign 1999*

**Table 3** Relative risks (ORs) of CIN according to smoking history, marital status, reproductive history and sexual behaviour

| Variables | Number of cases/controls | Crude OR (95% CI) | OR adjusted for HPV (95% CI) |
|---|---|---|---|
| Cigarette smoking | | | |
| Never | 106/119 | 1.00 | 1.00 |
| Past | 13/17 | 0.91 (0.42–1.98) | 0.32 (0.08–1.27) |
| Current | 41/24 | 2.04 (1.06–3.91) | 0.81 (0.17–2.22) |
| Marital status | | | |
| Never-married | 12/23 | 1.00 | 1.00 |
| Married | 137/122 | 3.74 (1.36–10.32) | 7.32 (1.06–50.43) |
| Separated/widowed | 12/17 | 2.01 (0.59–6.91) | 1.16 (0.10–13.37) |
| Age at marriage | | | |
| –22 | 58/32 | 1.77 (1.01–3.12) | 3.13 (0.91–10.8) |
| 23–26 | 71/75 | 1.00 | 1.00 |
| 27+ | 24/34 | 0.66 (0.34–1.28) | 0.82 (0.23–2.90) |
| | *P* for trend | *P* = 0.005 | *P* = 0.25 |
| Number of pregnancies | | | |
| 0 | 8/23 | 0.46 (0.18–1.15) | 0.14 (0.02–1.03) |
| 1–2 | 39/91 | 1.00 | 1.00 |
| 3+ | 77/57 | 1.82 (1.03–3.20) | 2.72 (0.74–10.1) |
| | *P* for trend | *P* = 0.002 | *P* = 0.07 |
| Number of births | | | |
| 0 | 15/28 | 0.57 (0.27–1.19) | 0.26 (0.05–1.23) |
| 1–2 | 81/91 | 1.00 | 1.00 |
| 3+ | 54/38 | 1.75 (1.01–3.03) | 3.21 (1.03–10.0) |
| | *P* for trend | *P* = 0.002 | *P* = 0.002 |
| Age at first pregnancy | | | |
| –23 | 76/46 | 1.84 (1.02–3.32) | 4.42 (1.17–16.7) |
| 24–26 | 42/47 | 1.00 | 1.00 |
| 27+ | 34/42 | 0.83 (0.44–1.58) | 1.75 (0.53–5.79) |
| | *P* for trend | *P* = 0.001 | *P* = 0.14 |
| Age at first birth | | | |
| –24 | 70/43 | 1.76 (0.99–3.14) | 1.31 (0.44–3.90) |
| 25–27 | 45/51 | 1.00 | 1.00 |
| 28+ | 23/35 | 0.62 (0.30–1.28) | 0.38 (0.08–1.82) |
| | *P* for trend | *P* = 0.002 | *P* = 0.06 |
| Use of oral contraceptives | | | |
| Never | 152/151 | 1.00 | 1.00 |
| Ever | 10/12 | 0.91 (0.39–2.14) | 0.93 (0.16–5.34) |
| Age at first intercourse | | | |
| –18 | 19/17 | 0.85 (0.38–1.91) | 0.11 (0.02–0.71) |
| 19–23 | 114/94 | 1.00 | 1.00 |
| 24+ | 30/53 | 0.50 (0.30–0.84) | 0.53 (0.21–1.35) |
| | *P* for trend | *P* = 0.03 | *P* = 0.74 |
| Lifetime number of sexual partners | | | |
| 0–1 | 70/93 | 1.00 | 1.00 |
| 2–3 | 54/44 | 1.90 (1.11–3.30) | 0.94 (0.36–2.43) |
| 4+ | 38/27 | 2.19 (1.12–4.30) | 0.92 (0.26–3.22) |
| | *P* for trend | *P* = 0.01 | *P* = 0.88 |

early age at first birth was associated with an enhanced risk of high-grade CIN (Cuzick et al, 1990). This factor was found to be independent of multiparity and marital status in the multivariate analysis. Thus, it is conceivable that the uterine cervix of young pregnant women, mostly in concert with HPV, is more vulnerable to the development of CIN.

Being married was found to be a HPV-independent risk factor for CIN in this study. This factor was firstly described as a risk factor of cervical cancer in Rigoni-Stern's study in 1842 (Rotkin, 1973). It is to be noted that marital status was a risk factor independent of multiparity in the multivariate analysis.

It is of interest that smoking was no longer a risk factor of CIN after adjustment for HPV infections as in several recent studies (Schiffman et al, 1993; Olsen et al, 1995). Other lines of research have documented smoking as a risk factor for high-grade CIN (Kjaer et al, 1998; Olsen et al, 1998), and the present data do not rule out the possibility that smoking is a risk factor for CIN progression.

Lifetime number of sexual partners and age at first intercourse turned out not to be risk factors after controlling for HPV infection. This suggests that sexual behaviour per se may be a risk factor of HPV infection but not CIN, in keeping with previous studies (Schiffman et al, 1993; Becker et al, 1994).

*© Cancer Research Campaign 1999*

**Table 4** Relative risks (ORs) for CIN according to selological variables

| Variables | Number of cases/controls | Crude OR (95% CI) | OR adjusted for HPV (95% CI) |
|---|---|---|---|
| HSV (IgG) | | | |
| Negative | 33 46 | 1.00 | 1.00 |
| Positive | 125/116 | 1.67 (0.97–2.86) | 2.03 (0.70–5.89) |
| HSV (IgM) | | | |
| Negative | 148/155 | 1.00 | 1.00 |
| Positive | 10/7 | 1.50 (0.53–4.21) | 1.05 (0.19–5.78) |
| CMV (IgG) | | | |
| Negative | 5/11 | 1.00 | 1.00 |
| Positive | 153/151 | 2.20 (0.76–6.33) | 9.66 (0.77–120.9) |
| CMV (IgM) | | | |
| Negative | 152/157 | 1.00 | 1.00 |
| Positive | 6/4 | 1.67 (0.40–6.97) | 1.19 (0.09–15.7) |
| CT (IgA) | | | |
| Negative | 119/137 | 1.00 | 1.00 |
| Positive | 39/25 | 1.94 (1.08–3.49) | 1.65 (0.60–4.51) |
| CT (IgG) | | | |
| Negative | 121/141 | 1.00 | 1.00 |
| Positive | 37/21 | 2.07 (1.12–3.83) | 1.14 (0.41–3.15) |

Thus far, a limited number of studies have carried out serological analyses for sexually transmitted agents in the search of risk factors of CIN. Certain studies found an independent role of antibodies against CT or CMV as risk factors for high-grade CIN (Koutsky et al, 1992; de Sanjose et al, 1994). Here we demonstrated that the serological markers of HSV, CMV and CT were not HPV-independent risk factors for CIN as in another recent study (Ferrera et al, 1998).

In the present study, we focused on the analysis of risk factors in the development of CIN. The results yield clues toward elucidating the mechanism of the development of CIN in conjunction with HPV. It is to be noted that risk factors for the progression of CIN to cervical cancer may be different from those for CIN development. To elucidate aetiologic factors for CIN progression, a cohort study is underway.

### ACKNOWLEDGEMENTS

We thank Prof. Y Taketani, University of Tokyo, for his critical review. This work was supported by grants from the Ministry of Education, Science, Sports and Culture of Japan.

### REFERENCES

Bartholomew JS, Glenville S, Sarkar S, Burt DJ, Stanley MA, Ruiz-Cabello F, Chengang J, Garrido F and Stern PL (1997) Integration of high-risk human papillomavirus DNA is linked to the down-regulation of class I human leucocyte antigens by steroid hormones in cervical tumor cells. *Cancer Res* **57**: 937–942

Becker TM, Wheeler CM, Mcgough NS, Stidley CA, Parmenter CA, Dorin MH and Jordan SW (1994) Contraceptive and reproductive risks for cervical dysplasia in southwestern Hispanic and non-Hispanic white women. *Int J Epidemiol* **23**: 913–930

Bornstein J, Rahat MA and Abramoviei H (1995) Etiology of cervical cancer: current concepts. *Obstet Gynecol Survey* **50**: 146–154

Chen YH, Huang LH and Chen TM (1996) Differential effects of progestins and estrogens on long control regions of human papillomavirus types 16 and 18. *Biochem Biophys Res Commun* **224**: 651–659

Cuzick J, Singer A, De Stavola BL and Chomet J (1990) Case-control study of risk factors for cervical intraepithelial neoplasia in young women. *Eur J Cancer* **26**: 684–690

de Sanjos S, Munoz N, Bosch FX, Reimann K, Pedersen NS, Orfila J, Ascunce N, Gonzalez LC, Tafur L, Gili M, Lette I, Viladiu P, Tormo MJ, Moreo P, Shah K and Wahren B (1994) Sexually transmitted agents and cervical neoplasia in Colombia and Spain. *Int J Cancer* **56**: 358–363

de Villiers, EM, Wagner D, Schneider A, Wesch H, Miklaw H, Wahrendorf J, Papendick U and zur Hausen H (1987) Human papillomavirus infections in women with and without abnormal cervical cytology. *Lancet* **ii**, 703–706

Ferrera A, Baay MF, Herbrink P, Figueroa M, Velema JP and Melchers WJ (1998) A seroepidemiological study of the relationship between sexually transmitted agents and cervical cancer. *Int J Cancer* **73**: 781–785

Kjaer SK, van den Brule AJ, Svare EI, Engholm G, Sherman ME, Poll PA, Walboomers JM, Bock JE and Meijer CJ (1998) Different risk factors high-grade and low-grade intraepithelial neoplasia on the cervix among HPV-positive and HPV-negative young women. *Int J Cancer* **75**: 613–619

Koutsky LA, Holmes KK, Critchlow CW, Stevens CE, Paavonen J, Beckmann AM, DeRouen TA, Galloway DA, Vernon D and Kiviat NB (1992) A cohort study of the risk of cervical intraepithelial neoplasia grade 2 or 3 in relation to papillomavirus infection. *New Engl J Med* **327**: 1272–1278

Lorincz AT, Reid R, Jenson B, Greenberg MD, Lancaster W and Kurman RJ (1992) Human papillomavirus infection of the cervix: relative risk associations of 15 common anogenital types. *Obstet Gynecol* **79**: 328–337

Olsen AO, Gjoen K, SAuer T, Orstavik I, Naess O, Kierulf K, Sponland G and Magnus P (1995) Human papillomavirus and cervical intraepithlial neoplasia grade II-III: a population-based case-control study. *Int J Cancer* **61**: 312–315

Olsen AO, Dillner J, Skrondal A and Magnus P (1998) Combined effect of smoking and human papillomavirus type 16 infection in cervical carcinogenesis. *Epidemiology* **9**: 346–349

Rotkin ID (1973) A comparison review of key epidemiological studies in cervical cancer related to current searches for transmissible agents. *Cancer Res* **33**: 1353–1367

Schiffman MH, Bauer HM, Hoover RN, Glass AG, Cadell DM, Rush BB, Scott DR, Sherman ME, Kurman RJ, Wacholder SK, Stanton CK and Manos MM (1993) Epidemiologic evidence showing that human papillomavirus infection causes most cervical intraepithelial neoplasia. *J Natl Cancer Inst* **85**: 958–964

Yoshikawa H, Kawana T, Kitagawa K, Mizuno M, Yoshikura H and Iwamoto A (1991) Detection and typing of multiple genital human papillomaviruses by DNA amplification with consensus primers. *Jpn J Cancer Res* **82**: 524–531

zur Hausen H (1991) Human papillomaviruses in the pathogenesis of anogenital cancer. *Virology* **184**: 9–13

*© Cancer Research Campaign 1999*
